# Live cell optical super-resolution microscopy of dystroglycan mutants as a model for dystroglycanopathies in multiple cell lines

**DOI:** 10.3389/fmolb.2025.1558170

**Published:** 2025-04-03

**Authors:** Francesca Sciandra, Manuela Bozzi, Alina Witt, Paul Goffing, Sonia Covaceuszach, Sandra Blaess, Alberto Cassetta, Maria Giulia Bigotti, Thomas Huser, Andrea Brancaccio, Wolfgang Hübner

**Affiliations:** ^1^ Institute of Chemical Sciences and Technologies “Giulio Natta” (SCITEC)-CNR, Rome, Italy; ^2^ Dipartimento di Scienze biotecnologiche di base, Cliniche intensivologiche e perioperatorie, Sezione di Biochimica e Biochimica Clinica, Catholic University of the Sacred Heart, Rome, Italy; ^3^ Department of Physics, Bielefeld University, Bielefeld, Germany; ^4^ CNR - Istituto di Cristallografia – Sede Secondaria di Trieste, Trieste, Italy; ^5^ Neurodevelopmental Genetics, Institute of Reconstructive Neurobiology, Medical Faculty, University of Bonn, Bonn, Germany; ^6^ Bristol Heart Institute, Research Floor Level 7, Bristol Royal Infirmary, Bristol, United Kingdom; ^7^ School of Biochemistry, Faculty of Health and Life Sciences, University of Bristol, Bristol, United Kingdom

**Keywords:** muscular dystrophy, dystroglycan, subcellular localization, super-resolution fluorescence microscopy, missense mutation, molecular diagnostics, dystroglycanopathies

## Abstract

**Introduction:**

Dystroglycan (DG) is an adhesion complex comprising two subunits, α-DG and β-DG, which interact non-covalently at the plasma membrane. As a component of the dystrophin-glycoprotein complex DGC, DG plays a crucial role in linking the cytoskeleton to the surrounding basement membranes. Rare primary point mutations in the *DAG1* gene have been identified in patients with various forms of neuromuscular dystrophy, ranging in phenotype from mild to severe.

**Methods:**

To gain a deeper understanding of the molecular mechanisms underlying these pathologies, we have designed a series of chimeric GFP-tagged full-length α/β-DG constructs and expressed them in three different cell lines (U-2OS, HEK-293T and C2C12). Wild-type DG constructs were compared to their counterparts carrying pathologic missense mutations previously described in patients, namely, L84F, T190M and C667F and with the mutant I591D, i.e., the topological equivalent of V567D identified in zebrafish.

**Results:**

Live super-resolution fluorescence microscopy showed that the C667F mutant is retained within the ER/Golgi while the T190M and wild-type proteins are correctly localized to the plasma membrane in all 3 cell lines. The L84F mutant exhibits a delay in trafficking to the plasma membrane in two of the cell lines, while localizing strongly at the plasma membrane in the high-expression HEK-293T cells. Similarly, the I591D mutant accumulated at the plasma membrane in the HEK-293T cells, in contrast to the clear retention in the endoplasmic reticulum/Golgi apparatus observed in U-2OS and C2C12 cells.

**Discussion:**

Our data demonstrate the importance of using a range of different cell lines for a comprehensive study of DG mutants or variants by live cell optical super-resolution microscopy.

## 1 Introduction

Dystroglycan (DG) is a ubiquitously expressed transmembrane protein complex formed by two subunits, α- and β-DG, encoded by a single gene (*DAG1* in humans) (Ibraghimov-Beskrovnaya et al., 1992). DG is expressed as a precursor peptide which undergoes a series of maturation steps initiated by the cleavage into its two subunits. α-DG is heavily glycosylated and, together with β-DG, forms an adhesion complex located at the plasma membrane. In skeletal muscle, DG is part of the dystrophin-glycoprotein complex (DGC), a multi-subunit protein complex that links the actin cytoskeleton to the extracellular matrix (Ervasti and Campbell, 1993; Liu et al., 2025; Wan et al., 2025). The DGC plays a crucial role in maintaining the mechanical stability of skeletal muscle fibers during the contraction-relaxation cycles. Consequently, the DG complex is fundamental for sarcolemma stability in skeletal muscle (Sciandra et al., 2003). Furthermore, DG plays a pivotal role in the assembly and stability of basement membranes at neuromuscular junctions and within the central nervous system, as well as in developmental processes and T-cell activation (Sciandra et al., 2023).

Mutations in several components of the DGC result in the development of distinct forms of muscular dystrophy (MD), a group of genetic diseases characterized by progressive muscle weakness, degeneration, and necrosis (Sciandra et al., 2003). Several distinct forms of muscular dystrophy have been identified and characterized. For instance, Duchenne muscular dystrophy (DMD) is caused by mutations within the dystrophin gene, limb-girdle muscular dystrophies (LGMD), which are genetically heterogeneous, can be also caused by genetic alterations of sarcoglycans, and congenital muscular dystrophy type 1A (MDC1A) is due to mutations in the extracellular matrix protein laminin-211. Furthermore, genetic abnormalities of specific glycosyltransferases result in hypoglycosylated α-DG, which reduces the affinity of this protein towards laminin-211 and other extracellular matrix proteins (Michele et al., 2002). This characteristic is shared by some severe congenital muscular dystrophies (CMD), including Muscle-Eye-Brain disease and Walker-Warburg syndrome, as well as additional forms of LGMD (Riemersma et al., 2015). These diseases are collectively known as secondary dystroglycanopathies, in which the laminin-binding glycan scaffold protruding from α-DG (matriglycan) can harbor modifications affecting the ability of DG to interact with its binding partners (Yoshida-Moriguchi and Campbell, 2015).

To date, only a few point-mutations within the *DAG1* gene have been identified in patients affected by forms of muscular dystrophies classified as primary dystroglycanopathies (Brancaccio, 2019; Dempsey et al., 2019). The missense mutation L86F, which corresponds to L84F in murine DG, is located within the initial Ig-like subdomain in the α-DG N-terminal domain. It has been suggested that this mutation may be associated with a form of inherited arrhythmogenic cardiomyopathy when coupled with a mutation in PKP2, one of the five genes that comprise the essential adhesion structure in cardiomyocytes called cardiac desmosome (König et al., 2017). Since the pathogenicity of L84F/L86F has not yet been confirmed, it is more appropriate to define it as a variant. The homozygous missense mutation (c.575C>T), in which a threonine is replaced by methionine at the amino acid residue 192 (190 in the murine sequence) within the S6-like subdomain of the α-DG N-terminal domain, is associated with a mild form of limb-girdle muscular dystrophy (LGMD2P) and cognitive impairment (MDDGC9, MIM# 613818) (Hara et al., 2011). The T192M mutation does not affect the overall structure of the N-terminal domain. However, it alters the dynamic behavior of the entire α-DG N-terminal domain, and it is thought to impair its interaction with LARGE1, one of the key glycosyltransferases responsible for the correct glycosylation of the α-DG matriglycan-decorated domain (mucin-like domain) (Hara et al., 2011; Bozzi et al., 2015; Covaceuszach et al., 2017a; Covaceuszach et al., 2017b). Hypoglycosylation of α-DG has significant implications for DG function, weakening the connections between α-DG and the extracellular matrix components, particularly laminin, and reducing the ability of β-DG to form actin clusters (Palmieri et al., 2017).

The I591D mutation is mapped within the second Ig-like subdomain of the C-terminal domain of α-DG and corresponds to the V567D mutation observed in the zebrafish DG orthologue (Gupta et al., 2011). By means of computational analysis and the introduction of the I591D mutation in a recombinant peptide spanning the C-terminal domain of α-DG, we have demonstrated a local perturbation of the murine α-DG C-terminal domain, which could potentially affect the entire post-translational maturation of the protein (Pirolli et al., 2014). In fact, in zebrafish, the homozygous V567D mutant is depleted of DG in tissues and presents a severe muscular dystrophy with impaired locomotion behavior (Gupta et al., 2011).

Another homozygous missense mutation (c.2006G>T) was identified in two siblings affected by muscle-eye-brain disease with central nervous system anomalies (MIM# 616538) and multicystic leukodystrophy (Geis et al., 2013). This mutation results in a cysteine to phenylalanine substitution at residue 669 (667 in the murine sequence) in the extracellular domain of β-DG. *In vitro*, the C667F mutation has been shown to alter the folding of the recombinant β-DG ectodomain resulting in covalent oligomerization and impairing the correct post-translational processing of the DG precursor, which remains mostly engulfed in the endoplasmic reticulum (ER) (Signorino et al., 2018). In a mouse model, C667F resulted in a partially penetrant phenotype of embryonic lethality as well as myopathy and blood-brain barrier destabilization (Tan et al., 2024).

Although initial insights have been gained into the altered processing of DG in the context of these mutations, the molecular mechanisms underlying the pathological effects observed in primary dystroglycanopathies remain poorly understood. Available data suggest that defects in the DG-axis, resulting from α-DG hypoglycosylation or misfolding of the *DG* core protein, ultimately lead to significant disruptions in the organization and functional maintenance of the extracellular matrix, as well as in the preservation of a stable connection with the cytoskeletal framework (Brancaccio, 2019).

In this study, we present a live optical super-resolution microscopy approach employed to visualize and compare the distribution of DG WT complex and its mutants within subcellular compartments. The aim of this approach is to gain insight into the molecular mechanisms that underlie primary dystroglycanopathies. In particular, we focused on the four missense mutations in *DAG1* that are of clinical significance, namely, L84F, T190M, I591D, and C667F. To this end, a panel of different cell lines (HEK-293T, U-2OS and C2C12 cells) was transfected with a plasmid expressing full-length murine DG complex (WT or mutated) in fusion with green fluorescent protein (GFP) at its C-terminus. This approach allows for the labeling of both subunits in the α/β-precursor, but only β-DG in the cleaved protein. This made it possible to monitor and compare the processing, trafficking, and subcellular localization of WT DG and its mutated counterparts in three commonly used cell lines. Here, the behavior of the L84F and I591D mutants in HEK-293T cells appears significantly different than in U-2OS and C2C12 cells.

## 2 Materials and methods

### 2.1 Cell culture and transfection

All cell types were grown in DMEM (Gibco) with 10% fetal bovine serum (Sigma-Aldrich) supplemented with 1% penicillin/streptavidin and glutamine (Sigma-Adrich). Cells were seeded on #1.5H coverglass bottom ibidi chambered slides (µ-slide 8 well high glass bottom, catalog number #80807, ibidi GmbH, Germany) and transiently transfected with WT or mutant DG constructs using Lipofectamine3000 according to the manufacturer’s protocol (Invitrogen, Thermofisher, United States). Typically, 250 ng of vector DNA were used for each 1 cm^3^ ibidi slide well.

### 2.2 Plasmids

The single point mutations T190M, I591D and C667F were introduced into the murine DG construct containing a myc-tag inserted within the C-terminus of α-DG and cloned in pEGFP vector as described elsewhere (Morlacchi et al., 2012; Pirolli et al., 2014; Palmieri et al., 2017; Signorino et al., 2018). The L84F mutation was introduced in the same expression vector using the Quick Change site-directed mutagenesis kit (Agilent Technologies, Santa Clara, CA, United States) and the following primers:

Forward 5′-AGCATTCCAACGGATTTCATTGCCTCCAGTGGG-3′

Reverse 5′-CCCACTGGAGGCAATGAAATCCGTTGGAATGCT-3′

The following plasmids carrying a chimeric scarlet intrinsic fluorescent protein fused with an appropriate protein targeting a specific cellular compartment (AddGene, United States, https://www.addgene.org/) were also used, namely, vLamp1-mScarlet-I (#98827), specific for lysosome, Lck-mScarlet-I (#98821) specific for plasma membrane and mScarlet-I-LaminB (#98831), specific for the nuclear membrane.

### 2.3 Fluorescent microscopy: advanced and super-resolution (SR)

For steady-state images, live cells were imaged 20–24 h post transfections. Advanced diffraction limited fluorescent microscopy images were acquired on a DeltaVision Elite fluorescence deconvolution microscope with a LED excitation source filtered with 475/28 nm bandpass and emission recorded on a CoolSNAP HQ2 CCD (Photometrics, Tuczon AZ, United States) after a 528/48 nm filter. After 3D deconvolution, the images were stitched using the microscope provided SoftWorx v7 application (DeltaVision, Cytiva, United States). Super-resolution images were acquired from live cells kept for maximum time of 1 h at 23°C on a 3D structured illumination microscope (3D SR-SIM) OMX v4 (DeltaVision, Cytiva, United States). The eGFP images were acquired after excitation with a diode laser at 488 nm while mScarlet images required a 568 nm diode laser. The emissions were collected respectively by individual sCMOS cameras after 528/48 and 609/37 bandpass filters. Super-resolution images have a final maximum field of view of 40.96 µm × 40.96 µm. Z slices were recorded every 200 nm on the diffraction limited microscope while the 3D SR-SIM required 125 nm steps. Figures were initially designed in the omero.figures web module (Allan et al., 2012) where all image intensities were adjusted linearly for representation. The uniformity of the observed localizations per constructs was verified visually over the entire transfected surfaces. Representative images were selected from several recorded images per constructs transfected. Raw widefield, deconvolved, raw 3D-SIM and reconstructed super-resolution images are available upon request.

### 2.4 Immunoprecipitation and western blotting

Nineteen hours after transfection with WT and L84F DG-pEGFP contructs, U-2OS cells were harvested and lysed using the following buffer (PBS containing 1% TritonX-100 and proteinase inhibitors) for 1 h. After centrifugation at 10,000 rpm, the cleared protein extracts were quantified, and equal amount of total protein extracts were incubated with Myc-Trap^®^ Agarose beads (Chromotek) for immunoprecipitation. After several washes with washing buffer (10 mM TrisHCl pH 7.4 and 0.15 M NaCl), bound proteins were eluted with SDS-sample buffer. Proteins were resolved on a 4%–15% SDS-PAGE (Bio-Rad Laboratories, Hercules, CA, United States). Proteins were then transferred to nitrocellulose and probed with polyclonal sheep anti-human Dystroglycan (#AF6868, R&D System) followed by incubation with an anti-sheep antibody conjugated to HRP. The reactive bands were revealed using the luminol-based ECL system (Advansta, CA, United States).

## 3 Results

### 3.1 Widefield fluorescence microscopy of DG mutants in U-2OS cells

**FIGURE 1:**
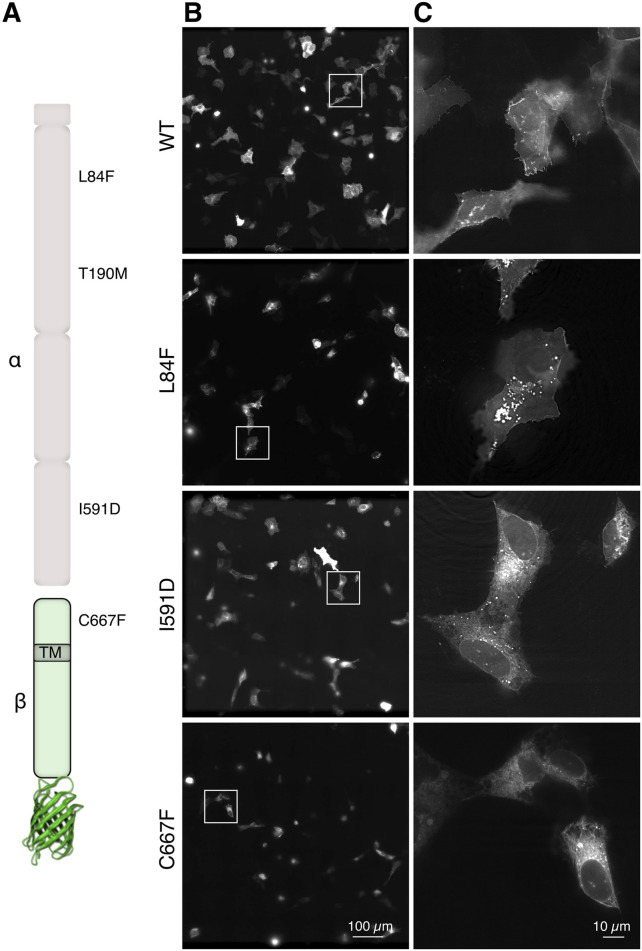
In-cell visualization of DG complex mutants by advanced fluorescence microscopy. **(A)** Schematic representation of the α-DG subunit (top, light pink) and the β-subunit fused to eGFP (bottom, light green). The locations of the 4 single missense mutations under investigation are indicated as well as the transmembrane domain (TM) location in the ß-DG subunit. **(B)** Three-dimensionally stitched 7 × 7 × 60 magnification field of views representing several U-2OS cells expressing the indicated DG complex variants. **(C)** Magnification of the cropped boxes from panels in **(B)**, which show the localization of DG in single cells.

We analyzed different DG point-mutations linked to dystroglycanopathies, namely, L84F, T190M, I591D and C667F (Figure 1A) that were introduced in the coding sequence of murine *dag1* cloned in a pEGFP-N1 vector (Pirolli et al., 2014; Palmieri et al., 2017; Signorino et al., 2018). In the pEGFP vector, gene expression is under the control of a highly permissive CMV promoter. In order to reduce any potential adverse effects resulting from the overexpression of GFP-constructs, we transfected human bone osteosarcoma epithelial cells (U-2OS), which exhibit a relatively lower transfection and expression efficiency. Moreover, these cells represent optimal “flat objects” for efficient illumination, particularly in 3D-SIM, as dense cells can diffract light, impeding the formation of an effective excitation pattern (Schermelleh et al., 2019). U-2OS cells were transfected transiently with plasmids encoding for eGFP fused to either WT or each of the aforementioned missense DG mutants (Figure 1A). Upon collection and imaging 20–24 h post-transfection at a high resolution with a ×60 magnification, deconvolved microscope images revealed distinct localizations (Figures 1B, C) of the variant proteins. The DG L84F mutant appears to have a similar subcellular localization at steady state to WT DG, with the majority of the protein accumulating at the plasma membrane and several bright internal vesicles. Notably the nuclear membrane appears visible indicating the possibility of increased ER accumulations. In contrast, both DG I591D and DG C667F strongly accumulate in the ER/Golgi network to a much greater extent than the wild-type protein (Signorino et al., 2018).

The diffraction-limited images, which lack sufficient optical sectioning and resolution, present a challenge in visualizing clear localization differences between the WT and the mutants. Notably with these flat cell culture, any cytoplasmic signal is overlayed by the plasma membrane.

### 3.2 Cell-type dependent subcellular localization of DG variants revealed by super-resolution fluorescence microscopy

**FIGURE 2:**
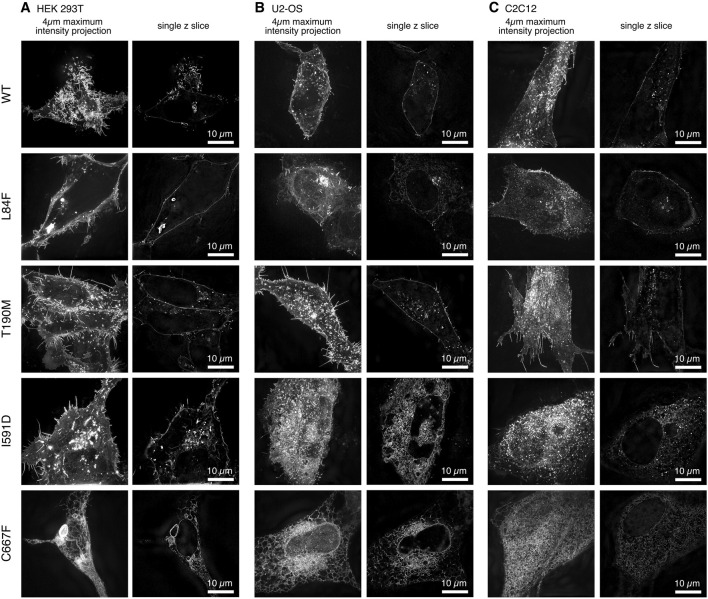
Cell line-specific subcellular localization of DG-eGFP fusion constructs visualized by 3D super-resolution microscopy. Three distinct cell lines were transfected with murine DG*-*eGFP: **(A)**. HEK-293T, **(B)**. U-2OS, and **(C)**. C2C12.

**FIGURE 3:**
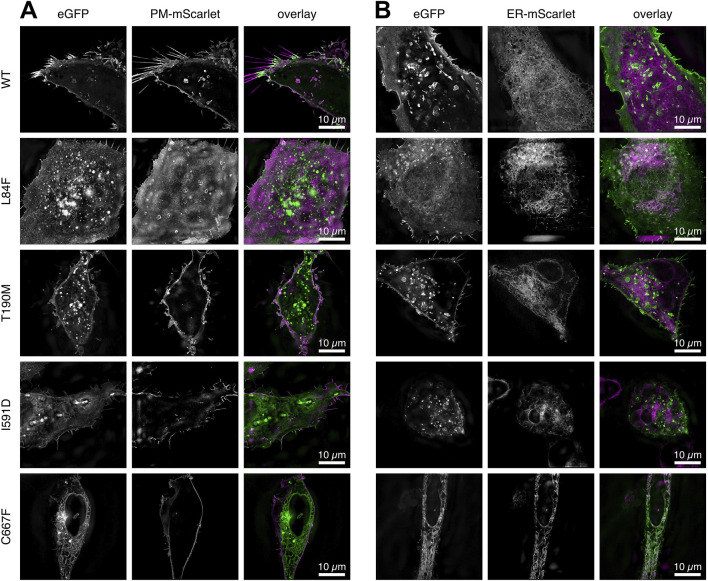
Two channel 3D SR-SIM of the WT DG and mutants complexes expressed in U-2OS cells. **(A)** Cells co-transfected with a plasma-membrane targeted mScarlet protein. **(B)** Cells co-transfected with an ER-targeted mScarlet protein. The signal accumulations in ER/Golgi for DG L84F, I591D and C667F correlate with the ER targeted fluorescent mScarlet protein. All images represent a 1 µm thick z-stack maximum intensity projection. DG L84F and I591D overlap with the plasma membrane localized mScarlet include the lower and upper portion of the cell, respectively, resulting in a diffuse plasma membrane signal. The individual channels eGFP for the DG complexes and plasma membrane or ER bound mScarlet (PM-mScarlet or ER-mScarlet) as well as the overlay in green and magenta respectively are shown.

The use of 3D super-resolution structured illumination microscopy to image WT and mutant DG-eGFP revealed differences in the localization of DG between cell types that were not clearly detectable with conventional diffraction-limited imaging (Figures 2, 3). The 2-fold increase in resolution in all dimensions also improved the optical sectioning and better defined the cytoplasmic signals.

In addition to the U-2OS cell line, we transiently transfected other commonly used cell lines, including human embryonic kidney HEK-293T and murine myoblasts C2C12. Interestingly we observed consistent differences in both the accumulation and localization of the DG complex between cell lines over time post-transfection (Figure 2).

In particular, in HEK-293T cells, at steady state, 20–24 h post-transfection, WT and DG T190M are predominantly localized at the plasma membrane and show a strong signal in internal vesicles. In contrast, DG I591D and DG C667F exhibit a strong intracellular signal, which correlates with the accumulation of the α/β-precursor in the ER/Golgi as well as in internal vesicles. It is noteworthy that DG C667F does not exhibit the same degree of vesicular accumulation as observed for DG I591D. Moreover, the mutant DG I591D appears to be differently distributed in the 3 cell types. In the HEK-293T cell line, DG I591D is clearly localized to the plasma membrane, a phenomenon that is not observed in the U-2OS or C2C12 cell lines (Figure 2).

The same discrepancies in expression and localization were observed in different cell lines for DG L84F. In HEK-293T, DG L84F appears to be localized primarily at the plasma membrane, exhibiting a similar distribution pattern to that of WT DG. In the U-2OS and C2C12 cell lines, the L84F DG complex signal is more nuanced and less dynamically distributed between the ER/Golgi apparatus and the plasma membrane (Figure 2). The DG L84F variant does not exhibit an overly high signal intensity at the plasma membrane compared to the ER/Golgi.

Finally, to further assess the subcellular localizations of WT and DG mutants in greater detail, double transfections of U-2OS cells were carried out with plasmids carrying membrane and ER scarlet-tagged markers, respectively (Figure 3).

### 3.3 Time-lapse recording of the DG-eGFP expression demonstrates the occurrence of intense trafficking activities and pathways

**FIGURE 4:**
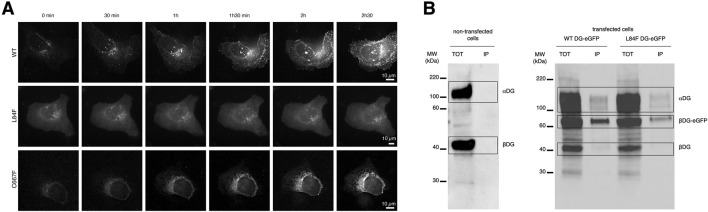
DG L84F complex proteins accumulate less efficiently at the plasma membrane with no apparent precursor cleavage defect. **(A)** Live imaging of U-2OS cells starting at 5 h post-transfection with eGFP- tagged WT DG, DG L84F, and DG C667F. 3D image stacks were captured every 30 min at ×60 magnification and deconvolved. **(B)** Total protein extracts of untransfected, WT-eGFP and L84F-eGFP transfected U-2OS cells were immunoprecipitated with anti-myc agarose beads. eGFP fusions constructs both carry a myc-tag within the C-terminal domain of α-DG and cloned into the pEGFP vector. Dystroglycan protein complex is detected by Western blot with a polyclonal sheep antibody that recognized both Dystroglycan subunits. No signal is detected in immunoprecipitated fraction from untransfected cells. For each immunoprecipitation blots, the total cellular (TOT) and the anti myc-tag immunoprecipitated (IP) fractions are shown. A broad band at 200 kDa reveals the α-subunit carrying the myc tag while β-DG fused to eGFP corresponds to the 60 kDa band. The endogenous β-DG subunit migrates at 40 kDa.

The dynamics of DG in vesicles/endosomes trafficking was recorded in U-2OS cells transfected with plasmids expressing WT DG, DG L84F, and DG C667F (Figure 4). The cells were imaged live 5 h post-transfection, as the fluorescent intensities increased in the cells. Initially, the GFP signal for all three proteins was observed in a region that appears to be exclusively the ER. Over the time, as the signal levels increased, WT DG is observed to predominantly localize at the plasma membrane. In contrast, DG C667F is found to accumulate in the ER while DG L84F exhibits more uniformly distributed localization to the ER, plasma membrane, and Golgi apparatus. Western blot analysis confirmed protein expression and the correct processing of the L84F precursor into the two DG subunits (Figure 4B). In contrast, DG C667F shows a significant deficiency in precursor processing (Signorino et al., 2018). Notably, DG L84F exhibits a delay in its maturation pathway 20–24 h post-transfection, as evidenced by its localization in the Golgi apparatus, unlike WT DG, which predominantly localizes to the plasma membrane (Figure 4A).

### 3.4 Overexpressed DG-GFP is targeted to lysosomes

The overexpression of the DG adhesion complex within U-2OS cells (driven by the strong CMV promoter) is likely to result in the presence of an overwhelming amount of DG-eGFP molecules within the cells, both in the intracellular membrane system, including the ER and the Golgi apparatus, and in other intracellular vesicular compartments such as exosomes and endosomes. In fact, the mature eGFP-tagged DG complex molecules (in which α-DG and β-DG are noncovalently linked) must be trafficked to the plasma membrane from the trans-Golgi network (TGN) via the secretory intracellular pathway (Hummer et al., 2020). Conversely, it is likely that the endolysosomal network will be involved in the potential recycling and degradation pathways triggered by the substantial quantities of the overexpressed DG adhesion complex.

**FIGURE 5:**
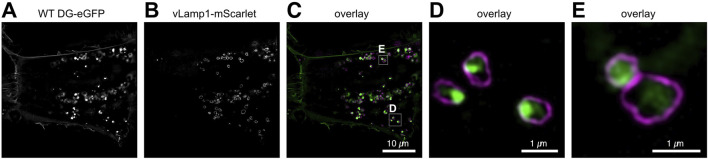
SR 3D-SIM imaging of the steady-state colocalization of WT DG-eGFP and lysosomes labeled with vesicular Lamp1 (vLamp1-mScarlet). **(A)** Individual channel for visualization of WT DG-eGFP. **(B)** Individual channel for visualization of vLamp1-mScarlet. **(C)** Overlay of the individual channels for WT DG-eGFP and vLamp1-mScarlet. **(D, E)** Magnification of the boxed areas in **(C)**. WT DG-eGFP complex is represented in green while vLamp1-mScarlet in magenta. Super-resolution microscopy reveals the presence of DG in late endosomes and lysosomes, which are defined by vesicular Lamp1 localization.

To gain insight into the trafficking of DG within the endolysosomal network, we co-transfected U-2OS cells with one vector expressing WT DG-eGFP and another expressing a vesicular Lamp1 mScarlet fusion protein (see Materials and Methods), which specifically labels lysosomal vesicles (Figure 5). The 3D SR-SIM images provide confirmation that lysosomes containing DG can be detected in these cells. Live-imaging reveals that several fast-moving lysosomal vesicles contain DG, although DG containing vesicles lacking the lysosomal marker are also observed (data not shown). It is noteworthy that the WT β-DG-GFP signal is predominantly observed within the lysosomal compartment. Nevertheless, it is important to consider that the signal may originate from GFP following the degradation of the entire complex within the lysosome. Additional experiments will be required to elucidate whether lysosomal DG is directed for degradation or for recycling back to the membrane.

### 3.5 β-dystroglycan is oriented towards the cytoplasmic side of the nuclear membrane

**FIGURE 6:**
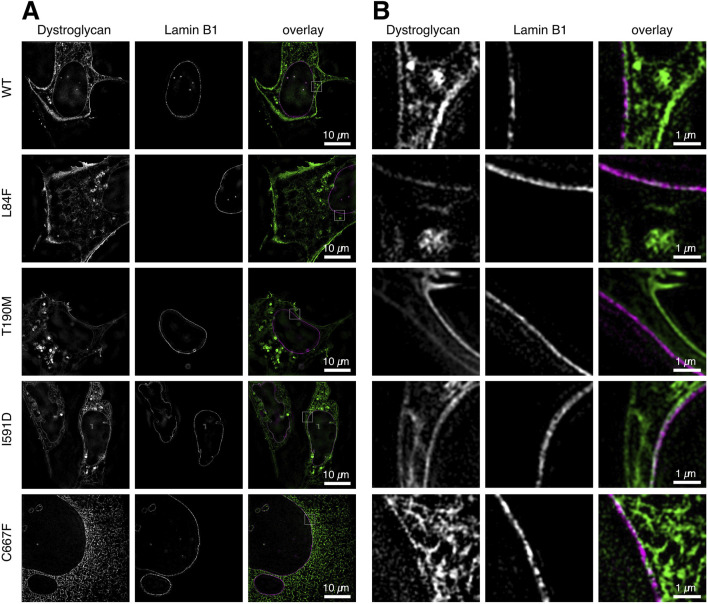
Co-localization analysis of WT and mutant DG-eGFP complexes with nuclear Lamin-B1 at the nuclear membrane. Steady-state localization of WT DG, DG L84F, DG T190M, DG I591D, and C667F mutant DG-eGFP are shown in U-2OS cells co-expressing nuclear Lamin-B1 labeled with the fluorescent protein mScarlet. **(A)** Single z-slice images of U-2OS cells co-expressing DG and nuclear Lamin-B1 labeled with eGFP and mScarlet, respectively, with a resolution of 40.96 µm × 40.96 µm. WT DG and DG T190M exhibits a strong localization at the plasma membrane and vesicular lysosomes while DG L84F, I591D and C667F show some retention in the ER. **(B)** Magnified view of the region of interest depicted in **(A)**. The individual channels are shown, as well as an overlay. The images were captured on live cells 20–21 h post-transfection using 3D super-resolution structured illumination.

The DG-eGFP fusion proteins carry a fluorescent protein at the C-terminus end of β-DG. During the translation, maturation, and transport of the exogenous proteins, the fluorescent signal is consistently present and appears first in the ER/Golgi. Even WT DG exhibits this signal 20–24 h after transfection, albeit at a very low intensity compared to the signal arising from its plasma membrane accumulation (Figure 4A). In the DG C667F mutant, the signal becomes progressively stronger in the ER/Golgi over time (Figures 4A, 6A, bottom row), indicating that it does not progress significantly towards the plasma membrane, in accordance with previous observations (Signorino et al., 2018). To determine the potential localization of these two proteins at the nuclear membrane, we conducted a co-transfection experiment in U-2OS cells. The cells were transfected with a plasmid for either WT DG or each of the mutants under investigation, fused to eGFP, and a vector expressing the nuclear lamina (NL) marker Lamin-B1 fused to the orange fluorescent protein mScarlet. In all cases, the eGFP signal appears in 3D SR-SIM as displaced and not overlapping with the inner nuclear membrane-associated Lamin-B1 (Figures 6A, B). This indicates a precise attachment of β-DG to the cytoplasmic side of the nuclear membrane, thereby confirming its association with the ER network rather than the nuclear membrane.

This could indicate a strict localization of β-DG bound to the cytoplasmic side of the nuclear membrane or a slight displacement of the mScarlet fluorophore of Lamin-B1 further into the nucleoplasm. Notably, these images are at the limit of the 3D SR-SIM technique, where such a small signal displacement is more likely to reflect the orientation of fluorophores to each other rather than the molecules to which they are covalently bound, making a definite interpretation of the data more challenging.

## 4 Discussion

DG follows a complex maturation pathway, that involves a series of post-translational modifications (Barresi and Campbell, 2006), trafficking, and targeting of the mature complex, from the ER/Golgi apparatus to the plasma membrane via exocytosis. Any alteration in the structure of the protein could, in principle, affect the subcellular localization of the two subunits, potentially impacting their biological function. Unfortunately the fixation strategies used for fluorescent imaging often result in significant modifications to cellular structures, including the ER and mitochondria. Therefore, rapid live-cell super-resolution microscopy was thus employed in this work to enable the efficient and reliable determination of the subcellular localization of WT DG and specific variants in various cell types.

In the cell lines employed, a high level of recombinantly expressed (exogenous) DG could be detected. The use of fluorescent eGFP allowed to track the DG complex and demonstrate how the spatial distribution of the detected signal could be altered in some missense DG mutants, particularly I591D and C667F, which are mainly expressed as uncleaved DG precursors (Pirolli et al., 2014; Signorino et al., 2018). Indeed, our findings demonstrated that these two variant DGs are engulfed in the ER/Golgi and are not properly delivered to the plasma membrane. This evidence has been corroborated in multiple cell lines, including the C2C12 myoblasts. Our data indicates that an impaired DG trafficking to the plasma membrane may have significant pathological consequences. The mouse model carrying the C667F mutation showed embryonic lethality and myopathy (Tan et al., 2024). These disorders could be due to two factors: firstly, the absence or significantly decreased levels of DG at the plasma membrane, which results in a reduction of the amount of α-DG available to recognize laminin molecules at the sarcolemma-basement membrane interface; secondly, the engulfment of intracellular compartments containing DG molecules, which may hyperactivate and overwhelm the ubiquitination and protein degradation pathways.

Like other mutations found in a heterozygous state in some patients (Dong et al., 2015), the L84F mutation is located in the Ig-like domain which is part of the N-terminal region of α-DG (König et al., 2017). This mutant was of particular interest due to its slightly altered behavior in different cell lines, as illustrated in Figure 2. To date, no biochemical or structural evidence has been uncovered that could link this variant to the observed pathology.

It is important to highlight that lower-resolution fluorescent microscopy techniques would be unable to reveal the localization phenotypes described in this study. With lower magnification measurements, the recorded focal plane is thicker, which can result in the mixing of signals at the plasma membrane with potentially weaker cytoplasmic signals. An even distribution of signal between the plasma membrane and the ER/Golgi, such as that observed in U-2OS and C2C12 cells for the DG L84F-eGFP mutant, would be challenging to visualize with this technique and would likely appear more similar to the WT distribution. The 3D super-resolution structured illumination microscopy method that we applied has the advantage to correct for small objects with low intensities by readjusting the linearity of the intensity distribution (Wu and Shroff, 2018). In addition, the resolution gain is two-fold in all dimensions, especially lateraly. This enables the visualization of the small low intensity ER/Golgi accumulations compared to the well-defined plasma membrane signal.

Another advantage of the method described here is the use of eGFP fusion expression systems. While the expression of a fluorescent protein fused to a protein of interest may affect its correct function and localization, eGFP is less susceptible to epitope and tag masking that is observed with other labeling methods (Hübner et al., 2007). For instance, antibody staining protocols involving proteins embedded in lipid membrane complexes may yield misleading intensity distributions, as epitopes may be masked in specific localization environments.

The observed rescue of localization defects of some variants when expressed in HEK-293T cells may be attributed to a more robust overexpression of the constructs or to differences in the trafficking pathways specific to this cell type. In the embryonic cell line HEK-293T, crucial components of the DGC such as sarcoglycans (Scano et al., 2024) are not expressed, which could greatly influence the stability of the dystroglycan complex (Liu et al., 2025; Wan et al., 2025). This could also impact in an unexpected way on the dynamic behavior of a mutant such as L84F, leading to the observed discrepancies in different cells lines. Consequently, our analysis strongly suggest the need to use multiple cell lines to study of the processing, trafficking, and targeting behavior of DG mutants and variants.

The techniques employed here allowed for the monitoring of DG complex trafficking towards the plasma membrane during its processing and synthesis (exocytosis), as well as some retrograde (plasma to intracellular) movements, represented by endocytic and lysosomal vesicles. This is a particularly noteworthy observation, as it suggests that this technique could be employed to analyze protein synthesis pathways and identify potential defects in vesicle behavior when novel mutations are identified. For example, the combined use of biosensors for autophagy such as fluorescent LC3 could clarify the trafficking mechanisms. Such insights could have significant diagnostic implications.

Furthermore, the localization of β-DG within the nucleus or at the nuclear membrane was examined in detail (Oppizzi et al., 2008; Vélez-Aguilera et al., 2018). The use of our panel of plasmids (WT and mutated) did not yield any discernible signal within the nuclear compartments of the U-2OS, HEK-293T, and C2C12 cell lines (see Figures 1–6). This phenomenon can be attributed to the presence of α-DG, which functions as a “driving force” that stabilizes β-DG at the plasma membrane. Conversely, a construct expressing the β-DG subunit alone [β-DG-GFP plasmid, (Lara-Chacón et al., 2010)] has been employed by others to confirm that, in addition to other intracellular compartments where β-DG is overexpressed (see Figure 6), a significant fraction of the β-DG subunit could indeed be localized at the inner nuclear membrane in U-2OS and HEK-293T cells, where it nearly co-localized with Lamin-B1.

Our comprehensive imaging approach on cell culture models demonstrates the subtle molecular and trafficking abnormalities in DG mutants associated with primary dystroglycanopathies. In particular, C667F and I591D represent paradigmatic cases in which the entire processing, maturation, trafficking, and targeting pathway is heavily impaired. The cellular models that we have established and investigated with these mutants may serve as useful model systems for testing pharmacological treatments aimed at rescuing the correct localization of mature DG or at reducing ER stress to reinstate a functional DG at the plasma membrane. These approaches may prove to be a valuable tool for the development of future therapeutic interventions.

We posit that, in addition to their value in elucidating the intricate maturation pathway of DG and its subunits within the cell, live-optical super-resolution microscopy of DG mutants in different cell lines could be proposed as a novel and powerful molecular diagnostic tool, especially in the future when the resolution limits are going to be pushed further. Ideally, it could complement routine antibody-based analyses of tissue biopsies, based on techniques such as immunofluorescence, immunohistochemistry and Western blotting. When novel DG mutants or variants are identified.

## Data Availability

The raw data supporting the conclusions of this article will be made available by the authors, without undue reservation.
